# Supplementary material for: Review and Meta-Analysis of the Evidence for Choosing between Specific Pyrethroids for Programmatic Purposes
Source: Insects. 2021 Sep 14;12(9):826. doi: 10.3390/insects12090826 (PMC8465213; doi:10.3390/insects12090826)
Supplement: Supplementary file 1 [file insects-12-00826-s001.zip › insects-1350040-supplementary.pdf]

Supplementary Material

Deltamethrin Dose Response Curves

A) CDC *An. albimanus* Teco

B) Cardiff *An. gambiae* G3

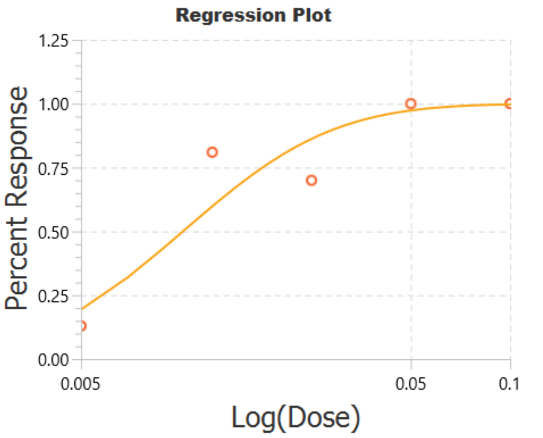

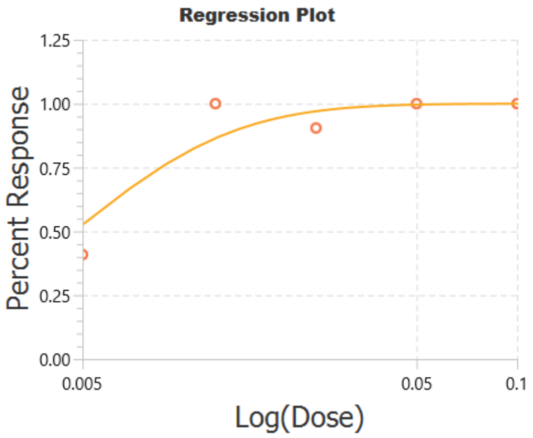

C) LIN *An. gambiae* G3

D) Mali *An. gambiae* Mopti

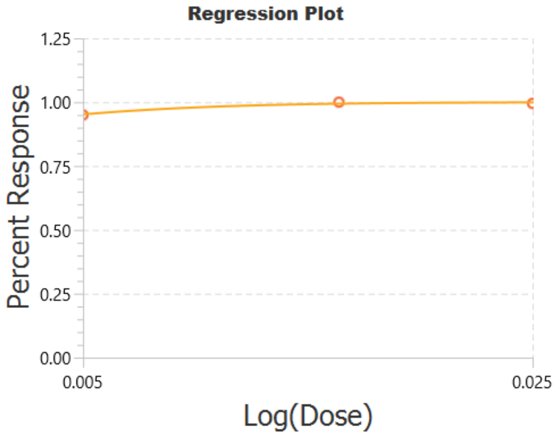

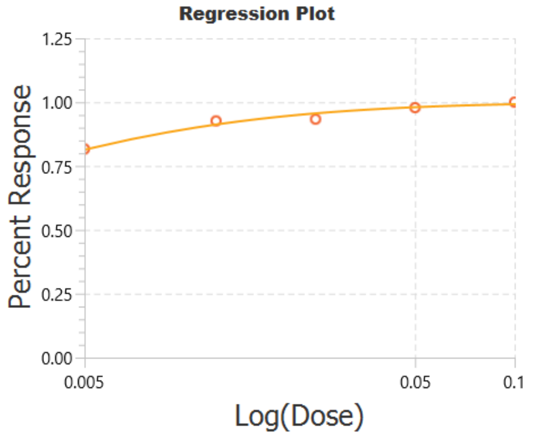

E) CDC *An. stephensi* Delhi

F) Cardiff *An. stephensi* St

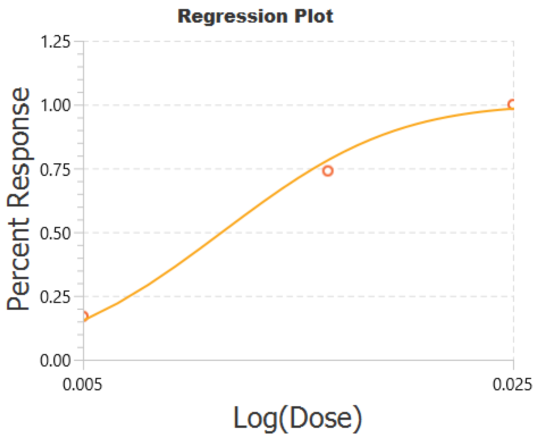

G) Cardiff *An. stephensi* Beech

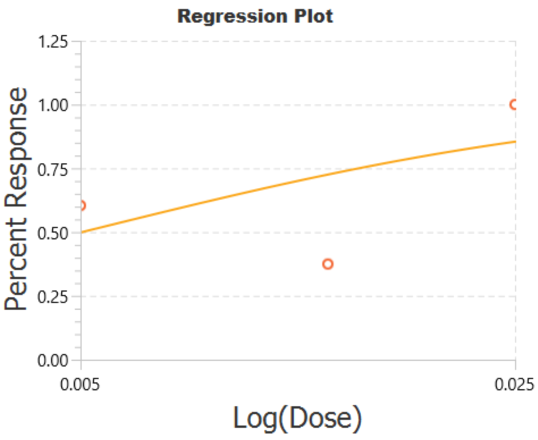

H) LSHTM *An. stephensi* Beech

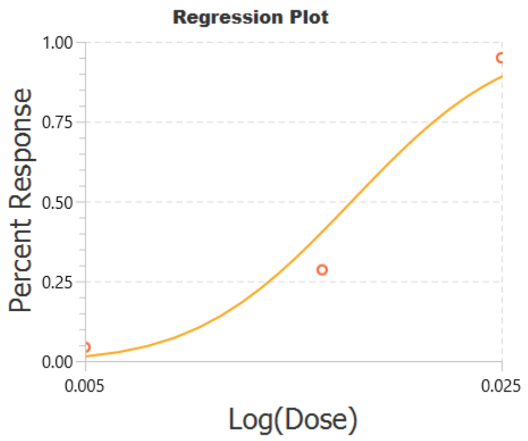

I) Iran *An. stephensi* Beech

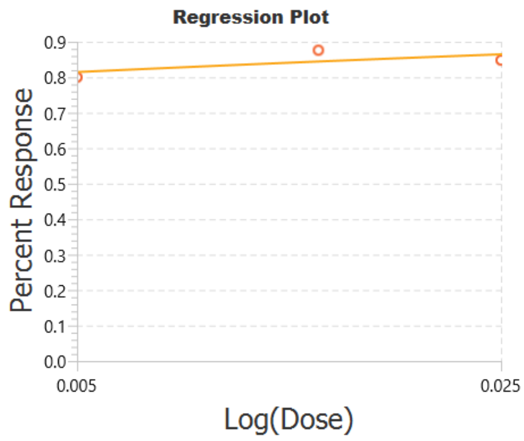

J) Pooled *An. albimanus*

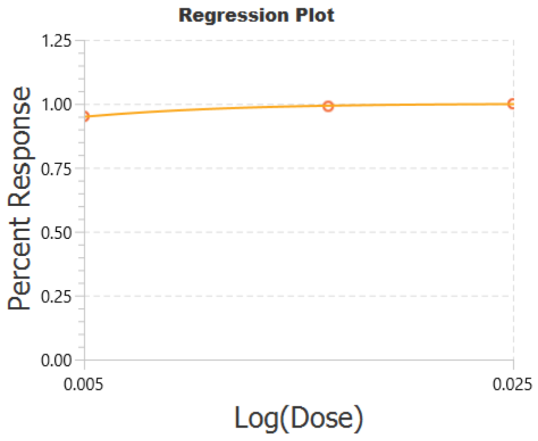

K) Pooled *An. gambiae*

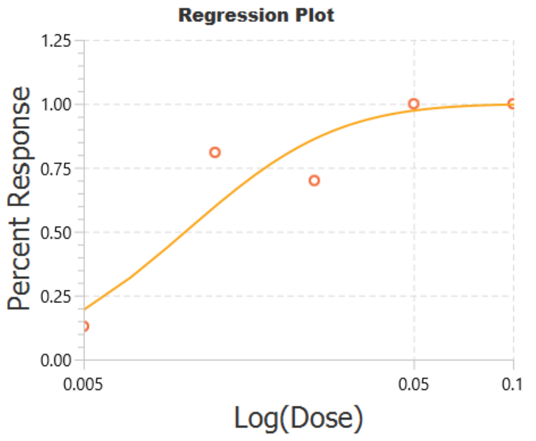

L) Pooled *An. stephensi*

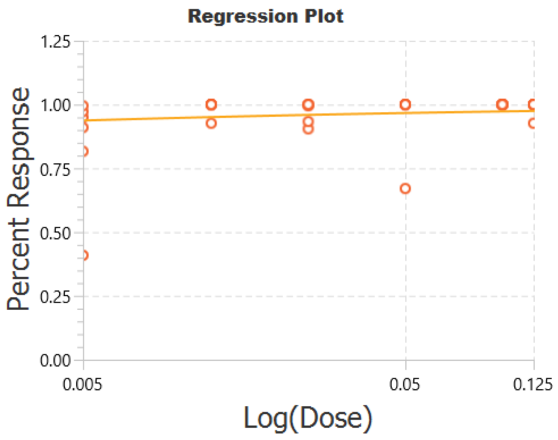

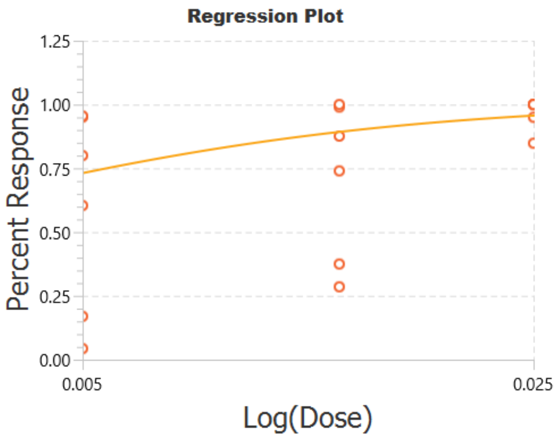

M) Deltamethrin pooled all data

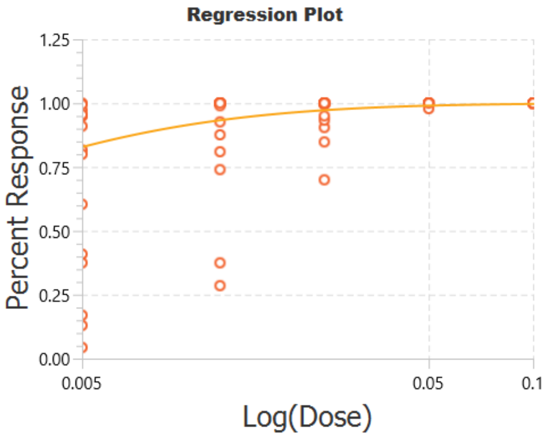

Permethrin Dose Response Curves

N) CDC *An. albimanus* Teco

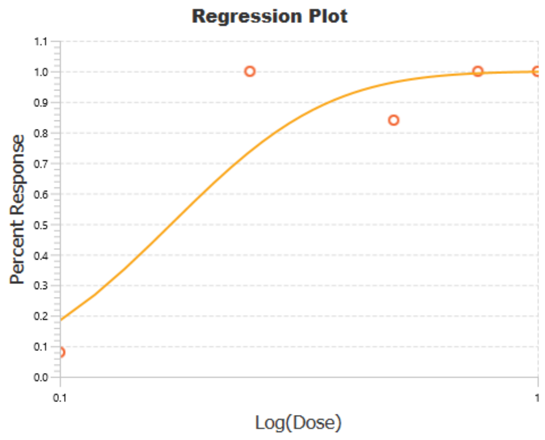

O) CDC *An. gambiae* G3

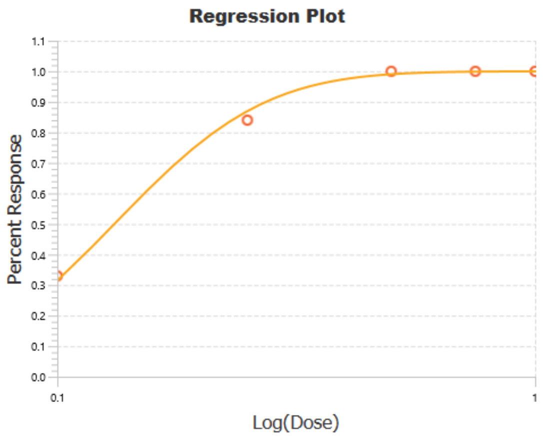

P) LIN *An. gambiae* G3

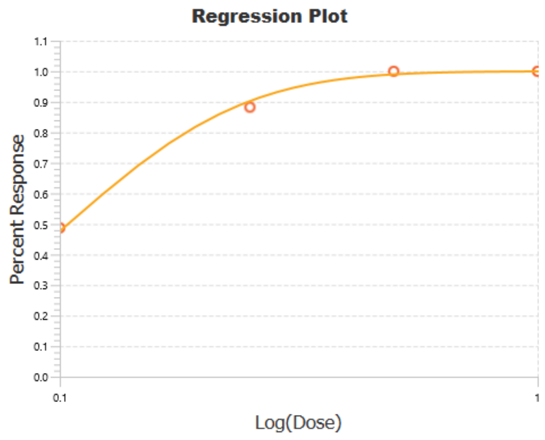

Q) LSHTM *An. gambiae* Kwa

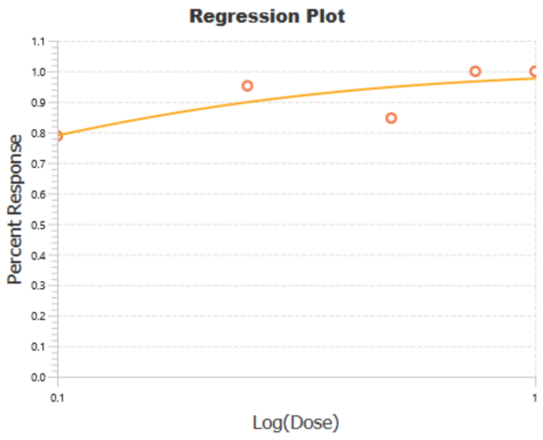

R) Mali *An. gambiae* Mopti

S) CDC *An. stephensi* Delhi

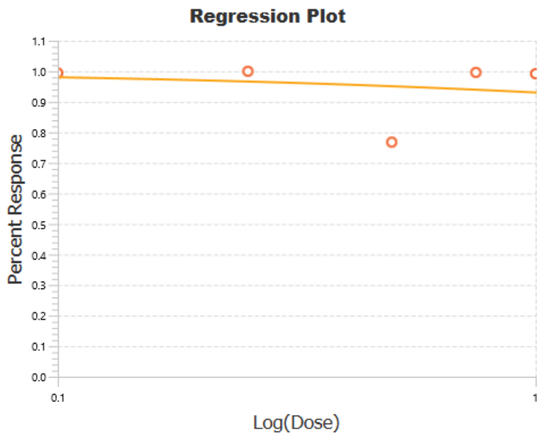

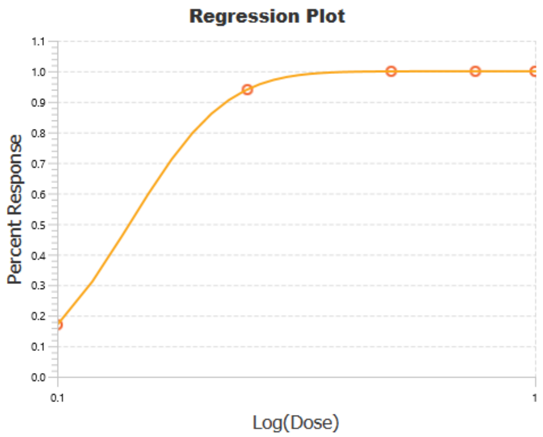

T) Cardiff *An. stephensi* Beech

U) LSHTM *An. stephensi* Beech

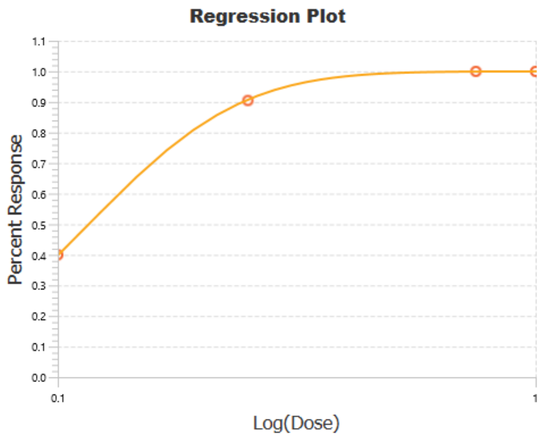

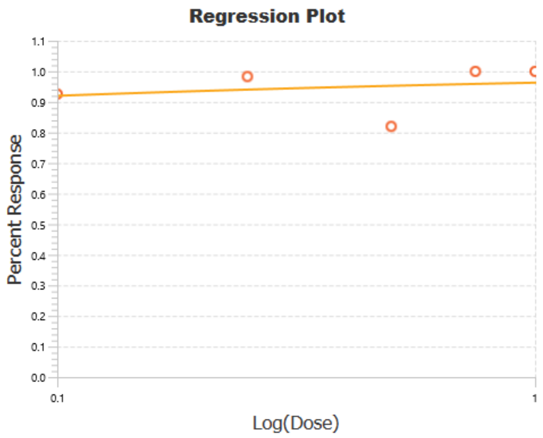

V) Pooled *An. albimanus*

W) Pooled *An. gambiae*

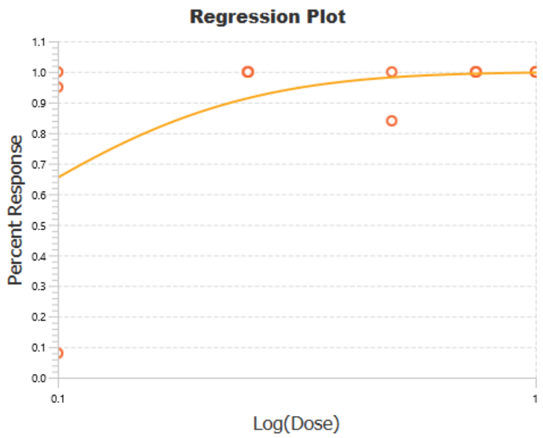

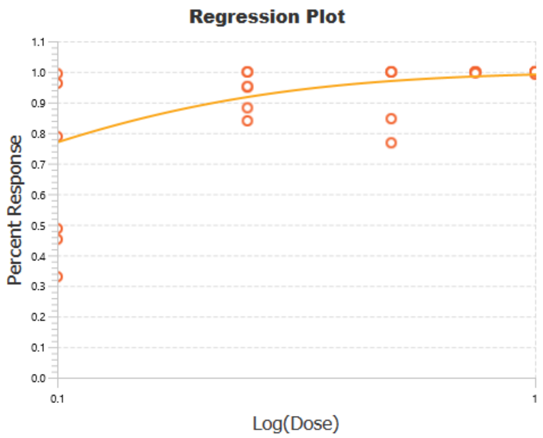

X) Pooled *An. stephensi*

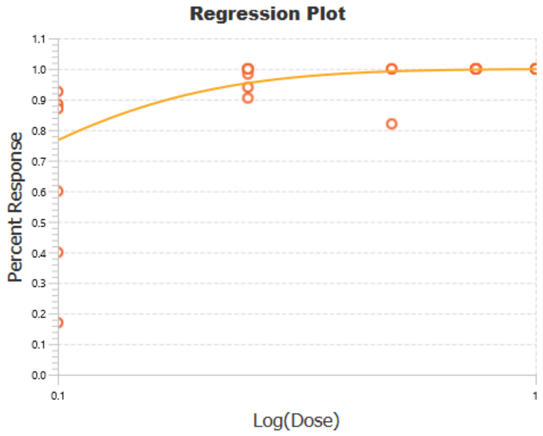

Y) Permethrin pooled all data

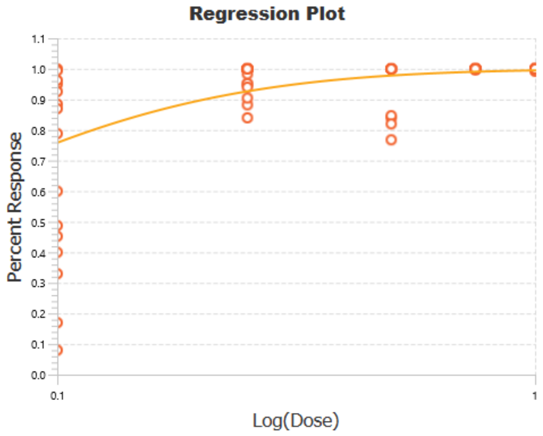

Figure S1. Dose response curves for deltamethrin (A – M) and permethrin (N – Y) using original raw data from the 1998 WHO multicentre study [8]

Table S1. Probit analysis of deltamethrin and permethrin using original raw data from the 1998 WHO multicentre study [8]. Analysis was conducted using PoloJR program within PoloSuite (Ver 2.1). The discriminating dose is twice the LD_{99}. Abbreviations: LD = Lethal dose, DD = Discriminating dose.

Institute	Species	Strain	Insecticide	No. concentrations test	Total Mosquitoes exposed	LD_{95}	LD_{95} 95% CI	LD_{99}	LD_{99} 95% CI	DD
**Deltamethrin**										
CDC	*An. albimanus*	Teco	Deltamethrin	5	500	0.04	0.017 - 0	0.07	0.025 - 0	0.14
Cardiff	*An. albimanus*	Panama	Deltamethrin	4	160	LD matrix not calculated	-	-	-	-
LSHTM	*An. albimanus*	Mexico	Deltamethrin	5	562	LD matrix not calculated	-	-	-	-
CDC	*An. gambiae*	G3	Deltamethrin	5	500	LD matrix not calculated	-	-	-	-
Cardiff	*An. gambiae*	G3	Deltamethrin	5	184	0.02	?	0.04	0.013 - 0.001	0.08
LIN	*An. gambiae*	G3	Deltamethrin	3	900	0	?	0.01	?	0.2
LSHTM	*An. gambiae*	Kwa	Deltamethrin	5	533	LD matrix not calculated	-	-	-	-
Mali	*An. gambiae*	Mopti	Deltamethrin	5	1525	0.02	0.013 - 0.061	0.08	0.038 -1.042	0.16
LIN	*An. gambiae*	Kisumu	Deltamethrin	4	1400	LD matrix not calculated	-	-	-	-

CDC	*An. stephensi*	Delhi	Deltamethrin	3	300	0.02	?	0.03	?	0.06
Cardiff	*An. stephensi*	St	Deltamethrin	3	120	0.06	?	0.17	?	0.34
Cardiff	*An. stephensi*	Beech	Deltamethrin	3	128	0.03	?	0.04	?	0.08
LSHTM	*An. stephensi*	Beech	Deltamethrin	3	317	1.72	?	347.81	0.015 - 0.008	695.62
Iran	*An. stephensi*	Beech	Deltamethrin	3	604	0.01	?	0.01	?	0.02
India	*An. stephensi*	Delhi	Deltamethrin	2	525	Excluded < 3 concentrations	-	-	-	-
Species pooled										
Pooled	*An. albimanus*	Pooled	Deltamethrin	-	-	0.03	0.013 - 22.116	0.06	0.022 - 10109.5039	0.12
Pooled	*An. gambiae*	Pooled	Deltamethrin	-	-	0.01	0.001 - 0.015	0.03	0.016 - 7.617	0.06
Pooled	*An. stephensi*	Pooled	Deltamethrin	-	-	0.02	0.01 - 0.001	0.06	0.018 - 0	0.12
All pooled										
Pooled	Pooled	Pooled	Deltamethrin	-	-	0.02	0.01 - 0.055	0.05	0.023 - 1.166	0.1

**Permethrin**										
CDC	*An. albimanus*	Teco	Permethrin	5	500	0.46	0.167 - 0.059	0.69	0.265 - 0.015	1.38
Cardiff	*An. albimanus*	Panama	Permethrin	4	160	LD matrix not calculated	-	-	-	-
LSHTM	*An. albimanus*	Mexico	Permethrin	5	504	LD matrix not calculated	-	-	-	-
CDC	*An. gambiae*	G3	Permethrin	5	500	0.34	0.275 - 0.457	0.5	0.38 - 0.76	1
Cardiff	*An. gambiae*	G3	Permethrin	4	162	0.25	0.242 - 0.258	0.36	0.34 - 0.372	0.72
LIN	*An. gambiae*	G3	Permethrin	4	1500	0.32	0.233 - 0.624	0.5	0.328 - 1.393	1
LSHTM	*An. gambiae*	Kwa	Permethrin	5	524	0.52	?	1.96	0.333 - 0.06	3.92
Mali	*An. gambiae*	Mopti	Permethrin	5	1425	0.52	?	0.04	?	0.08
LIN	*An. gambiae*	Kisumu	Permethrin	4	1400	LD matrix not calculated	-	-	-	-
CDC	*An. stephensi*	Delhi	Permethrin	5	500	0.26	0.252 - 0.264	0.33	0.321 - 0.34	0.66
Cardiff	*An. stephensi*	St	Permethrin	4	160	LD matrix not calculated	-	-	-	-
Cardiff	*An. stephensi*	Beech	Permethrin	4	162	0.3	0.275 - 0.335	0.45	0.393 - 0.52	0.9

LSHTM	*An. stephensi*	Beech	Permethrin	5	522	0.42	?	26.16	?	52.3 2
Iran	*An. stephensi*	Beech	Permethrin	5	1063	LD matrix not calculated	-	-	-	-
India	*An. stephensi*	Delhi	Permethrin	2	500	Excluded < 3 concentrations	-	-	-	-
Species pooled										
Pooled	*An. albimanus*	Pooled	Permethrin	-	-	0.33	0.161 - 0	0.62	0.271 - 0	1.24
Pooled	*An. gambiae*	Pooled	Permethrin	-	-	0.36	0.218 - 1.518	0.93	0.445 - 21.446	1.86
Pooled	*An. stephensi*	Pooled	Permethrin	-	-	0.24	0.155 - 1.478	0.47	0.255 - 21.446	0.94
All pooled										
Pooled	Pooled	Pooled	Permethrin	-	-	0.32	0.233 - 0.544	0.73	0.452 - 2.067	1.46

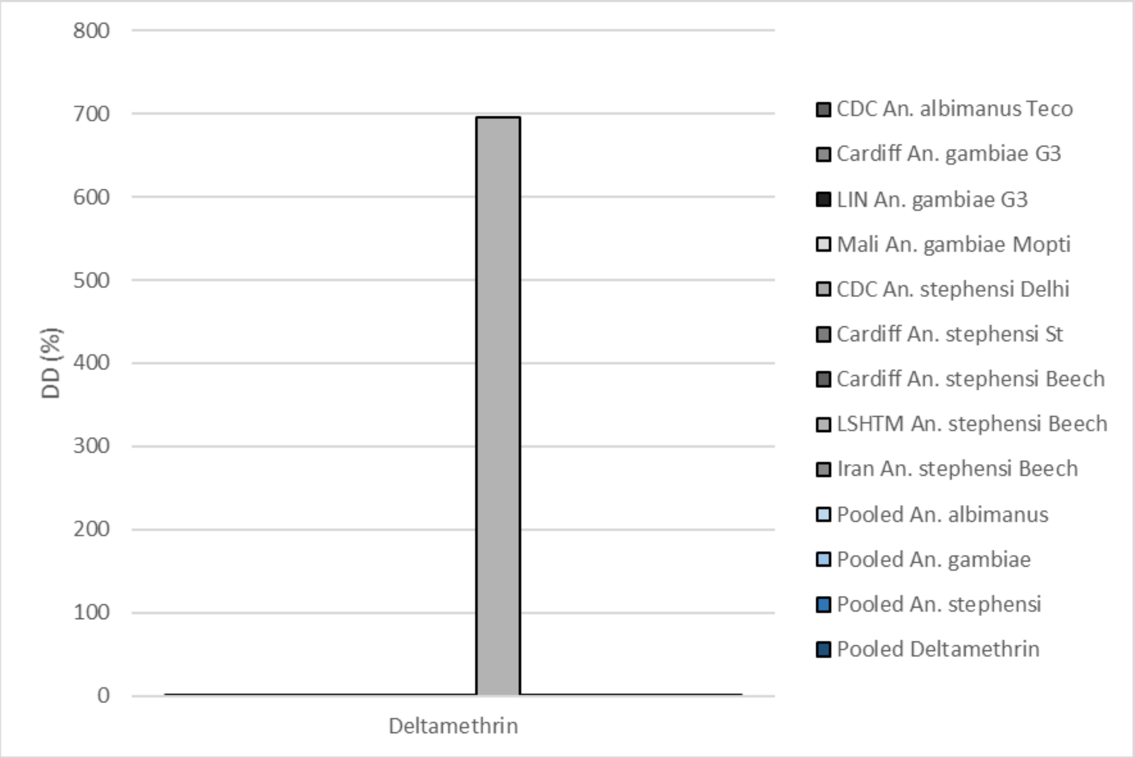

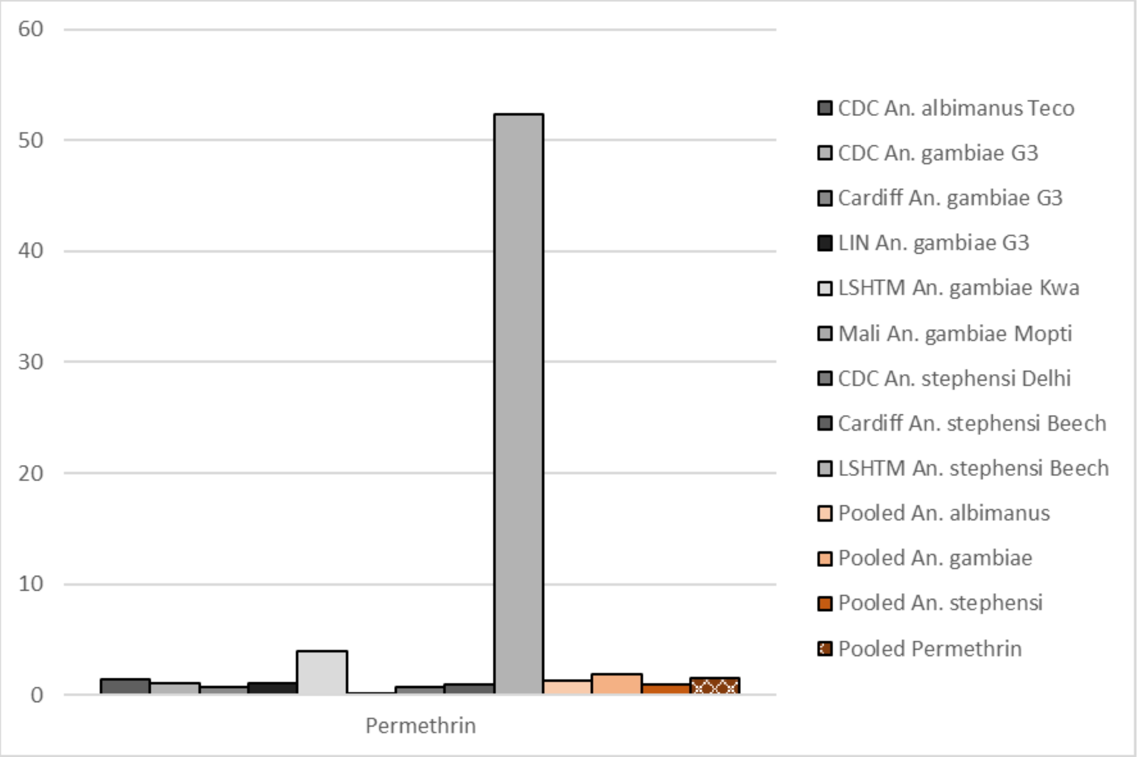

Figure S2. Calculated discriminating doses (%) for (**A**) deltamethrin and (**B**) permethrin. Points show individual sites/strain combinations, and data pooled by species or overall, by insecticide. Site/strain testing <3 concentrations of an insecticide and datasets not robust enough to calculate lethal dose matrixes are excluded. Discriminating doses are set at 2 x the calculated lethal dose at which 99% (LD99) of test mosquitoes were killed. LSHTM *An. stephensi* Beech data included.

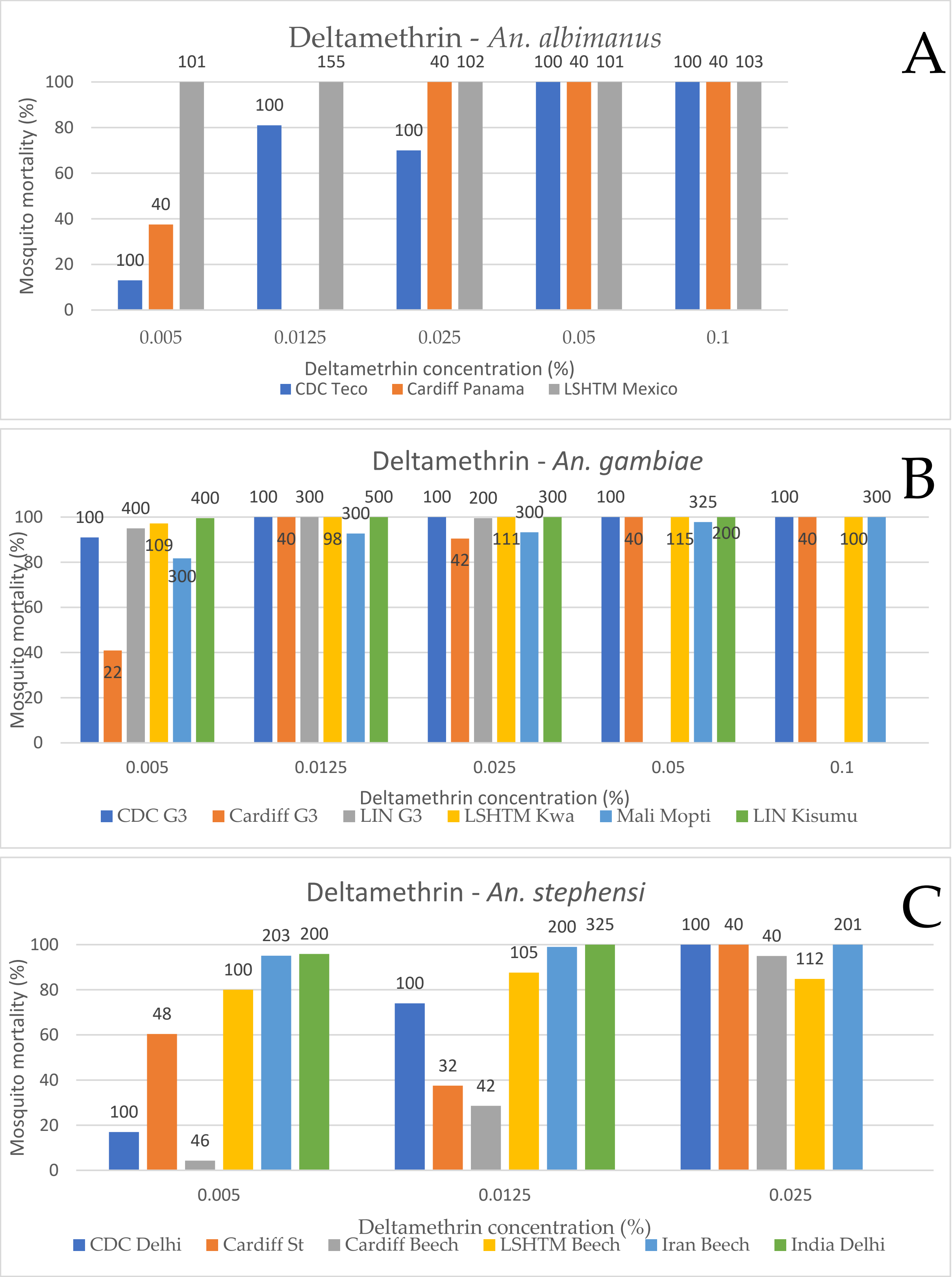

Figure S3. Mosquito mortality (%) following exposure to deltamethrin in WHO tube bioassays of site/strain combinations for (**A**) *An. albimanus*, (**B**) *An. gambiae*, and (**C**) *An. stephensi*. Numbers above bars show number of exposed mosquitoes.

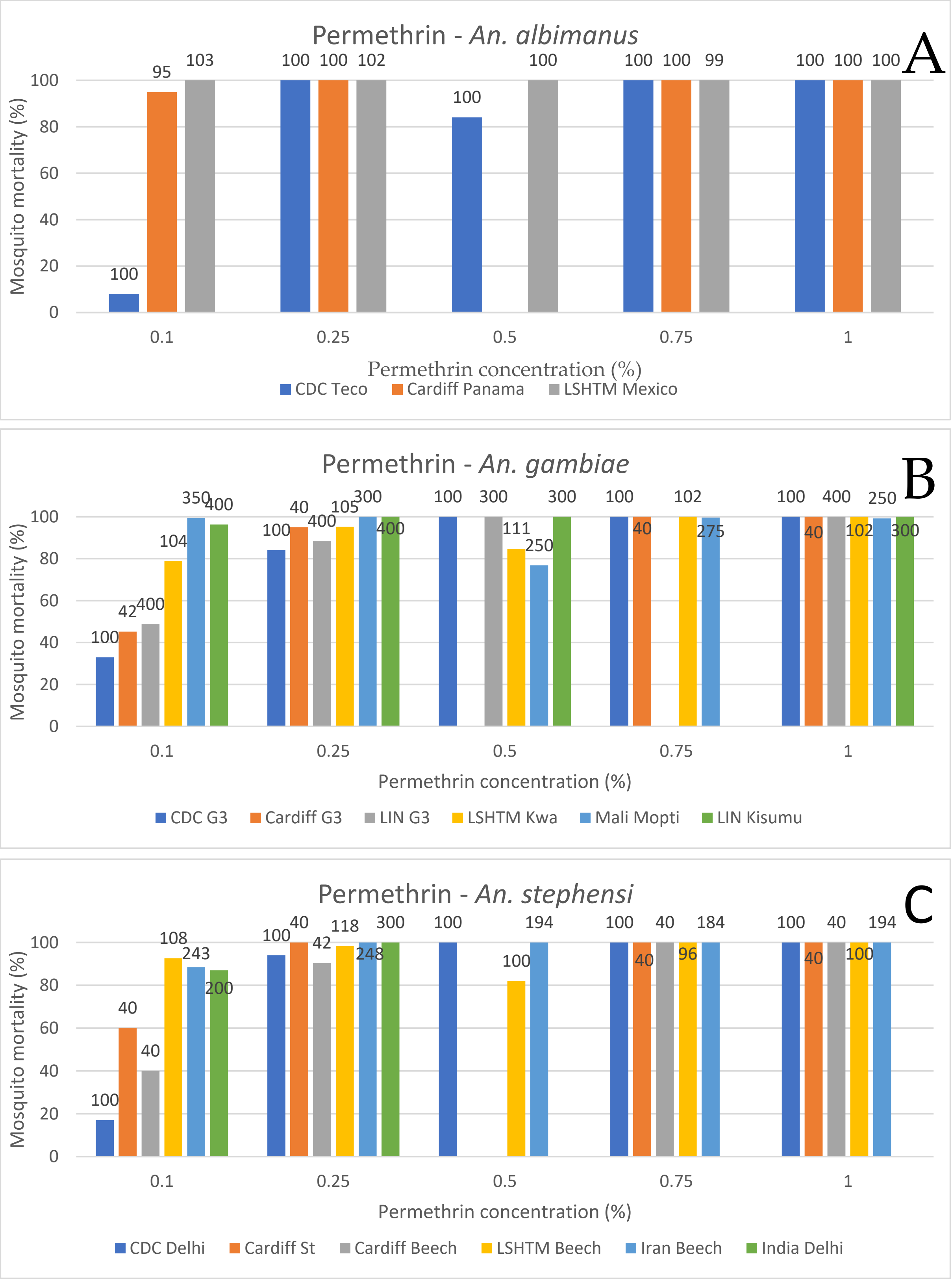

Figure S4. Mosquito mortality (%) following exposure to permethrin in WHO tube bioassays of site/strain combinations for (**A**) *An. albimanus*, (**B**) *An. gambiae*, and (**C**) *An. stephensi*. Numbers above bars show number of exposed mosquitoes.

Table S2. Summary statistics of variability in mosquito mortality following exposure to pyrethroids in standard WHO tube or CDC bottle bioassays. Mosquitoes were exposed to insecticides following the recommended methods for each test and mortality in each replicate tube or bottle was recorded 24-hours post-exposure. The strains detailed here are those maintained by the Ranson Group or LITE at LSTM, UK. Abbreviations: R = Insecticide resistant mosquito strain, S = Insecticide susceptible mosquito strain, IKR = inter-quartile range.

**Rearing group**	**Assay**	**Insecticide**	**Strain**	**R/S**	**Data points (replicate)**	**Lower range**	**Upper range**	**IKR**	**Mean**	**Median**	**Variance**	**Standard Deviation**
Ranson	Tube	α-cypermethrin	Kisumu *An. gambiae*	S	4	95.00	100.00	5.00	97.50	97.50	8.33	2.89
Ranson	Tube	Deltamethrin	Kisumu *An. gambiae*	S	4	100.00	100.00	0.00	100.00	100.00	0.00	0.00
Ranson	Tube	Permethrin	Kisumu *An. gambiae*	S	3	95.00	100.00	2.50	98.33	100.00	8.33	2.89
Ranson	Tube	α-cypermethrin	N'gousso *An. coluzzii*	S	8	90.00	100.00	5.00	97.50	100.00	14.29	3.78
Ranson	Tube	Deltamethrin	N'gousso *An. coluzzii*	S	4	100.00	100.00	0.00	100.00	100.00	0.00	0.00
Ranson	Tube	Permethrin	N'gousso *An. coluzzii*	S	8	84.21	100.00	0.00	98.03	100.00	31.16	5.58
Ranson	Tube	α-cypermethrin	Banfora-Sus *An. coluzzii*	S	4	92.00	100.00	5.00	95.00	94.00	14.67	3.83

Ranson	Tube	Deltamethrin	Banfora-Sus *An. coluzzii*	S	4	80.00	88.00	7.63	83.88	83.75	20.06	4.48
Ranson	Tube	Permethrin	Banfora-Sus *An. coluzzii*	S	4	68.00	92.31	13.14	79.39	78.62	114.05	10.68
Ranson	Tube	α-cypermethrin	Banfora M *An. coluzzii*	R	3	0.00	30.00	15.00	13.33	10.00	233.33	15.28
Ranson	Tube	Deltamethrin	Banfora M *An. coluzzii*	R	8	0.00	10.00	0.00	1.25	0.00	12.50	3.54
Ranson	Tube	Permethrin	Banfora M *An. coluzzii*	R	12	0.00	24.00	9.00	6.74	4.08	57.33	7.57
Ranson	Tube	α-cypermethrin	Bakaridjan *An. gambiae*	R	4	0.00	8.00	2.00	2.00	0.00	16.00	4.00
Ranson	Tube	Deltamethrin	Bakaridjan *An. gambiae*	R	8	0.00	7.69	4.81	2.40	0.00	12.42	3.52
Ranson	Tube	Permethrin	Bakaridjan *An. gambiae*	R	4	0.00	4.35	1.09	1.09	0.00	4.73	2.17
Ranson	Tube	α-cypermethrin	Gaoura *An. arabiensis*	R	4	0.00	8.00	2.00	2.00	0.00	16.00	4.00
Ranson	Tube	Deltamethrin	Gaoura *An. arabiensis*	R	8	0.00	4.00	4.00	2.00	2.00	4.57	2.14

Ranson	Tube	Permethrin	Gaoura *An. arabiensis*	R	8	0.00	12.00	2.00	2.50	0.00	22.57	4.75
Ranson	Tube	Deltamethrin	Tiefora *An. coluzzii*	R	8	0.00	7.69	4.00	2.46	2.00	8.39	2.90
Ranson	Tube	Permethrin	Tiefora *An. coluzzii*	R	8	0.00	7.69	4.38	3.09	3.92	8.00	2.83
Ranson	Tube	Deltamethrin	VK7 2014 *An. coluzzii*	R	4	4.17	13.04	3.72	8.80	9.00	13.85	3.72
Ranson	Tube	Permethrin	VK7 2014 *An. coluzzii*	R	4	0.00	8.33	5.65	5.06	5.95	16.42	4.05
Ranson	Tube	α-cypermethrin	Tiassale 13 *An. gambiae*	R	7	0.00	30.00	2.50	8.00	5.00	99.67	9.98
Ranson	Tube	Deltamethrin	Tiassale 13 *An. gambiae*	R	3	45.00	55.00	5.00	48.33	45.00	33.33	5.77
Ranson	Tube	Permethrin	Tiassale 13 *An. gambiae*	R	3	10.00	15.00	2.50	11.67	10.00	8.33	2.89
Ranson	Tube	α-cypermethrin	Fumoz *An. funestus*	R	4	0.00	4.00	1.00	3.00	4.00	4.00	2.00
Ranson	Tube	Deltamethrin	Fumoz *An. funestus*	R	24	0.00	16.67	8.08	4.68	4.00	22.06	4.70

Ranson	Tube	Permethrin	Fumoz *An. funestus*	R	6	0.00	28.00	3.07	6.62	3.85	113.40	10.65
LITE	Tube	α-cypermethrin	Fumoz *An. funestus*	R	4	0.00	10.00	3.10	5.08	5.16	16.78	4.10
LITE	Tube	Deltamethrin	Fumoz *An. funestus*	R	20	0.00	83.33	19.01	21.82	11.52	590.30	24.30
LITE	Tube	Permethrin	Fumoz *An. funestus*	R	23	0.00	86.21	42.67	25.24	9.52	847.92	29.12
LITE	Tube	α-cypermethrin	Kisumu *An. gambiae*	S	4	100.00	100.00	0.00	100.00	100.00	0.00	0.00
LITE	Tube	Deltamethrin	Kisumu *An. gambiae*	S	12	95.24	100.00	1.04	98.89	100.00	4.02	2.01
LITE	Tube	Permethrin	Kisumu *An. gambiae*	S	12	96.14	100.00	0.00	99.68	100.00	1.24	1.11
LITE	Tube	α-cypermethrin	Moz *An. arabiensis*	S	4	88.00	100.00	6.26	95.91	97.83	32.03	5.66
LITE	Tube	Deltamethrin	Moz *An. arabiensis*	S	12	83.33	100.00	13.00	93.19	96.00	46.13	6.79
LITE	Tube	Permethrin	Moz *An. arabiensis*	S	12	92.00	100.00	4.00	97.67	100.00	10.06	3.17

LITE	Tube	α-cypermethrin	Tiassale 13 *An. gambiae*	R	8	4.00	12.50	5.13	8.13	7.42	10.61	3.26
LITE	Tube	Deltamethrin	Tiassale 13 *An. gambiae*	R	24	0.00	41.67	12.51	15.35	12.25	121.23	11.01
LITE	Tube	Permethrin	Tiassale 13 *An. gambiae*	R	25	0.00	62.50	25.00	15.97	12.00	256.38	16.01
LITE	Tube	α-cypermethrin	VK7 2014 *An. coluzzii*	R	4	0.00	8.00	2.11	3.89	3.78	10.69	3.27
LITE	Tube	Deltamethrin	VK7 2014 *An. coluzzii*	R	16	0.00	13.04	5.13	3.49	0.00	23.06	4.80
LITE	Tube	Permethrin	VK7 2014 *An. coluzzii*	R	27	0.00	8.33	0.00	1.38	0.00	7.98	2.83
LITE	Bottle	Permethrin	Fumoz *An. funestus*	R	20	0.00	100.00	50.44	46.92	42.24	1121.92	33.50
LITE	Bottle	Permethrin + PBO	Fumoz *An. funestus*	R	18	69.57	100.00	0.00	96.62	100.00	96.87	9.84
LITE	Bottle	Permethrin	Kisumu *An. gambiae*	S	20	4.00	100.00	1.67	93.58	100.00	456.84	21.37
LITE	Bottle	Permethrin + PBO	Kisumu *An. gambiae*	S	17	8.00	100.00	0.00	94.59	100.00	497.88	22.31

LITE	Bottl e	Permethrin	Tiassale 13 *An. gambiae*	R	18	0.00	73.91	44.53	33.27	30.93	513.42	22.66
LITE	Bottl e	Permethrin + PBO	Tiassale 13 *An. gambiae*	R	21	4.17	100.00	16.67	88.62	100.00	454.27	21.31
LITE	Bottl e	Permethrin	VK7 2014 *An. coluzzii*	R	20	0.00	21.43	8.54	8.91	9.56	59.31	7.70
LITE	Bottl e	Permethrin + PBO	VK7 2014 *An. coluzzii*	R	20	25.00	100.00	7.73	87.06	100.00	630.80	25.12

Table S3. *p*-values (Welch's *t*-test) comparing mean mosquito mortality following exposure to α-cypermethrin 0.05%, deltamethrin 0.05%, or permethrin 0.75% in a standard WHO tube bioassay. Values significant at the 5% level ($p = < 0.05$) are highlighted in bold. Abbreviations; Delta = deltamethrin, Perm = permethrin, Alpha = α-cypermethrin.

Group	Strain	Delta v Perm	Delta v Alpha	Perm v Alpha	Delta v Perm + PBO	Perm v Perm + PBO	Alpha v Perm + PBO
Ranson	Banfora-Susceptible	0.482	**0.010**	0.055	-	-	-
	Kisumu	0.423	0.182	0.723	-	-	-
	N'gousso	0.351	0.104	0.829	-	-	-
	Banfora M	**0.044**	0.303	0.536	-	-	-
	Bakaridjan	0.446	0.870	0.706	-	-	-
	FUMOZ-R	0.680	0.254	0.451	-	-	-
	Gaoura	0.792	1.000	0.854	-	-	-

	Tiassalé 13	**0.002**	**0.000**	0.401	-	-	-
	Tiefora	0.668	-	-	-	-	-
	VK7 2014	0.223	-	-	-	-	-
LITE	Kisumu	0.251	0.082	0.339	-	-	-
	Moz	0.055	0.458	0.590	-	-	-
	FUMOZ-R	0.678	**0.009**	**0.004**	**0.000**	**0.000**	**0.000**
	Tiassalé 13	0.874	**0.008**	**0.029**	**0.002**	**0.001**	**0.002**
	VK7 2014	0.124	0.851	0.225	0.738	0.506	0.652

A) Banfora susceptible (Ranson group)

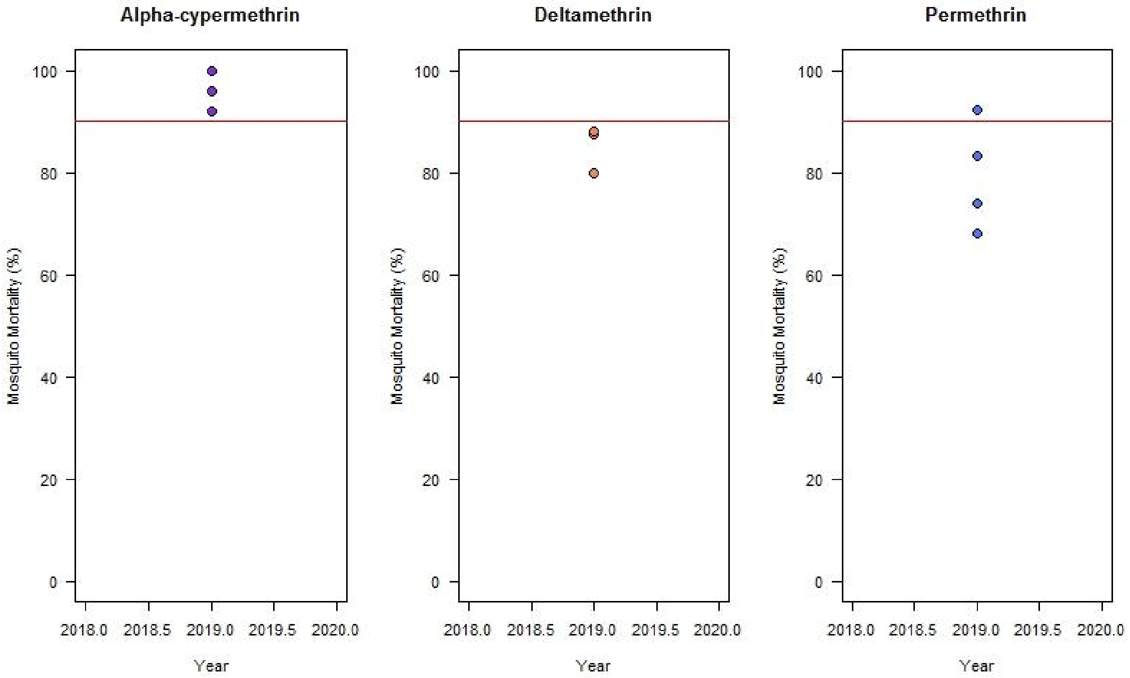

B) Kisumu (Ranson group)

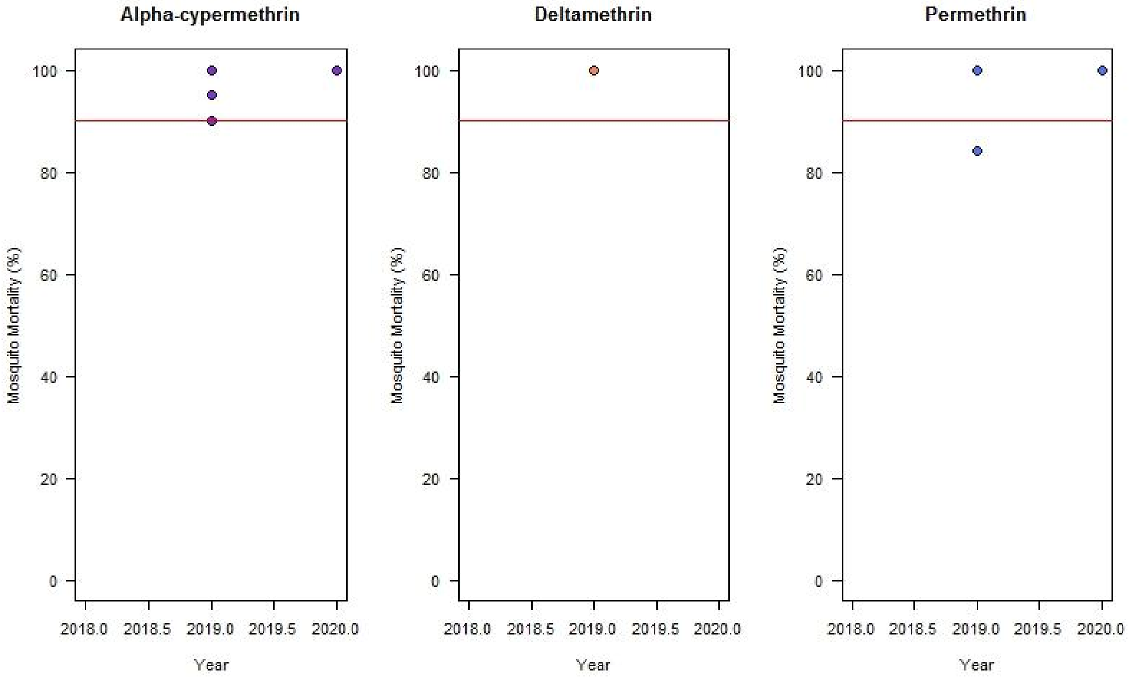

C) N’gousso (Ranson group)

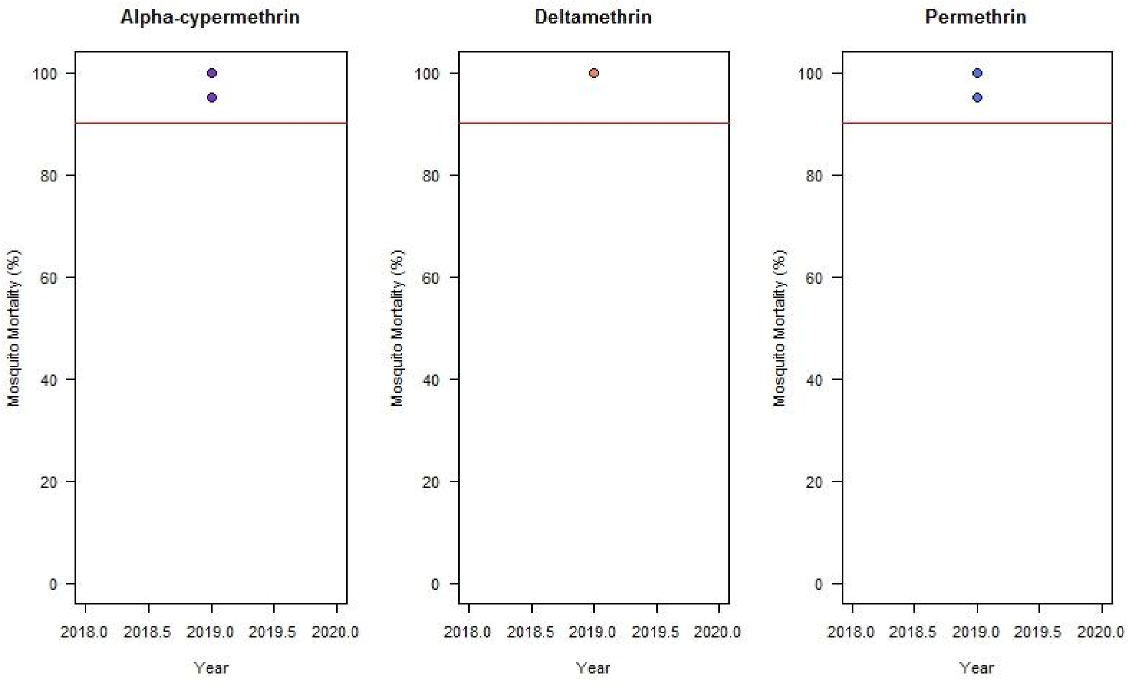

D) Banfora M (Ranson group)

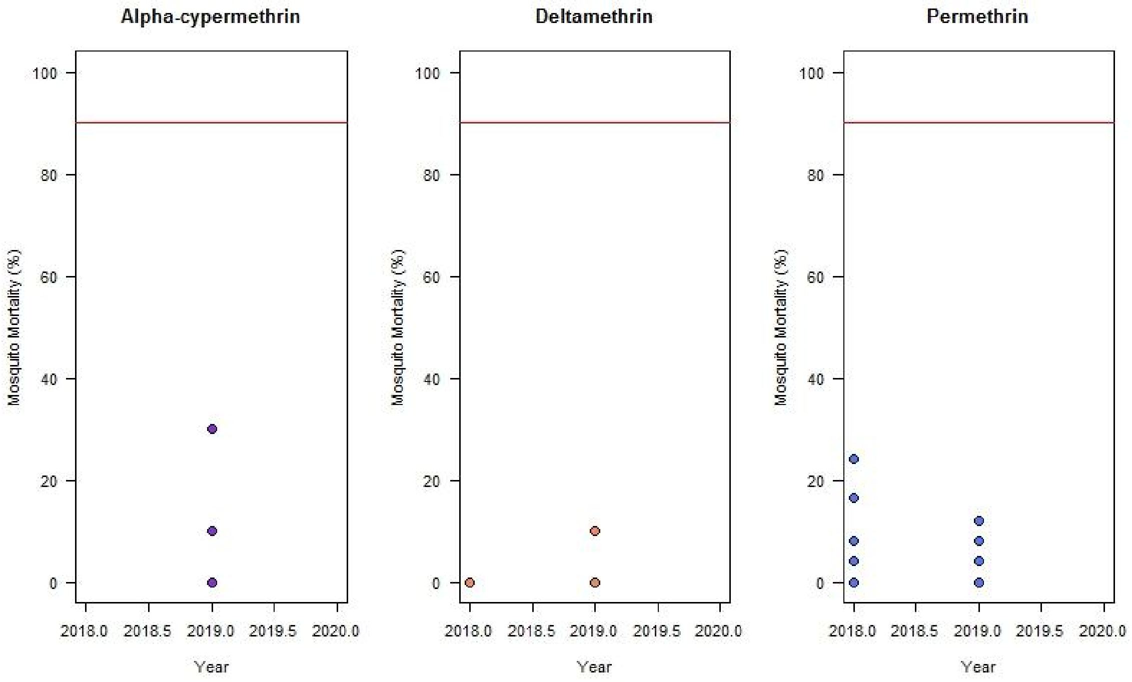

E) **Bakaridjan (Ranson group)**

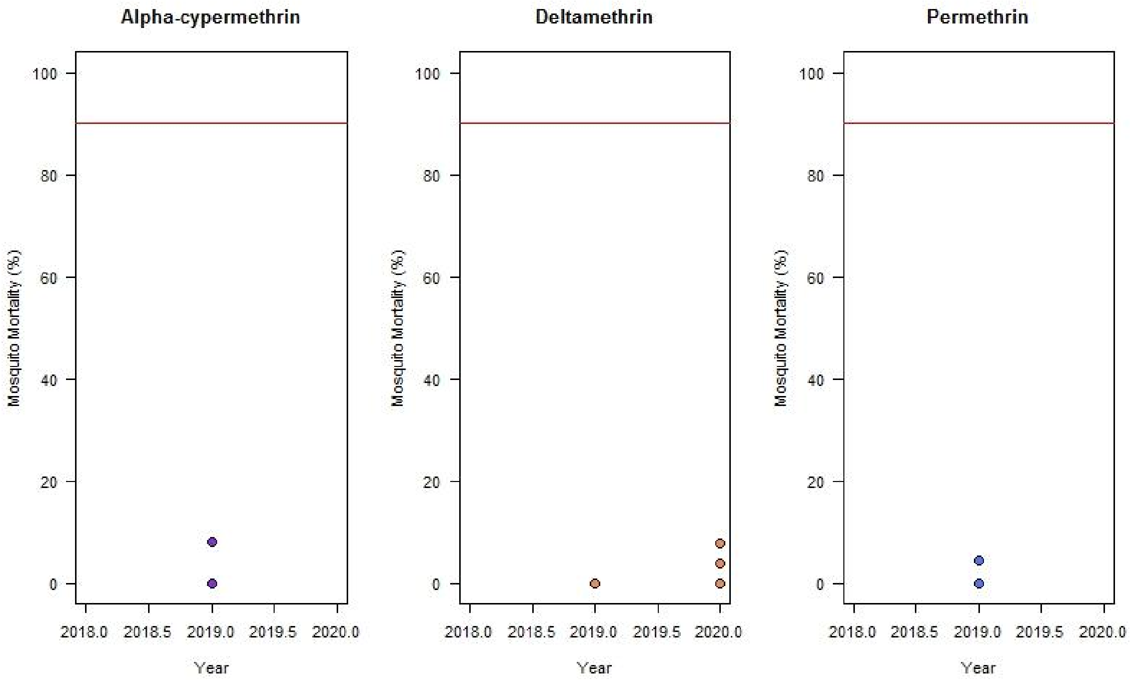

F) **FUMOZ-R (Ranson group)**

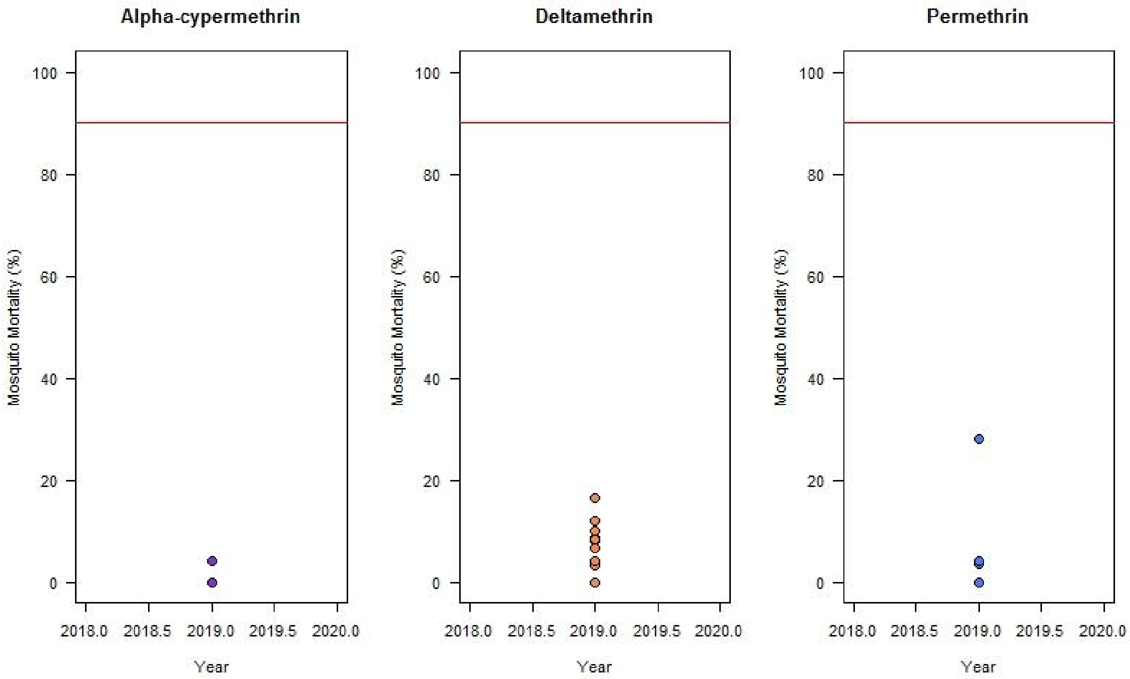

G) Gaoura (Ranson group)

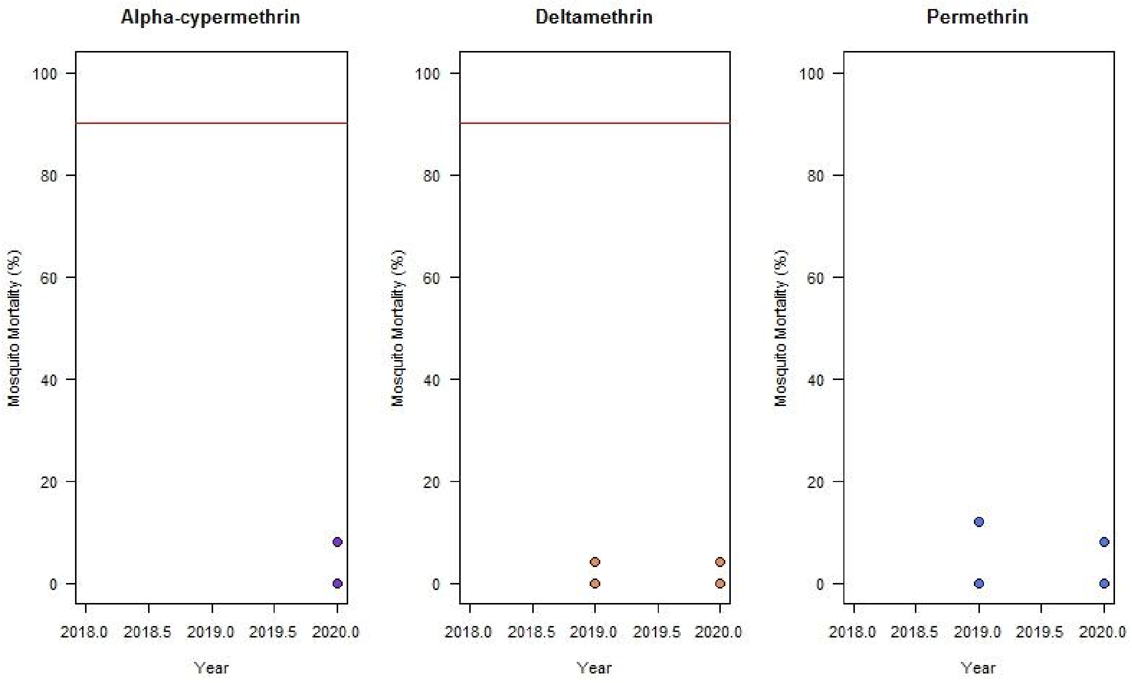

H) Tiassalé 14 (Ranson group)

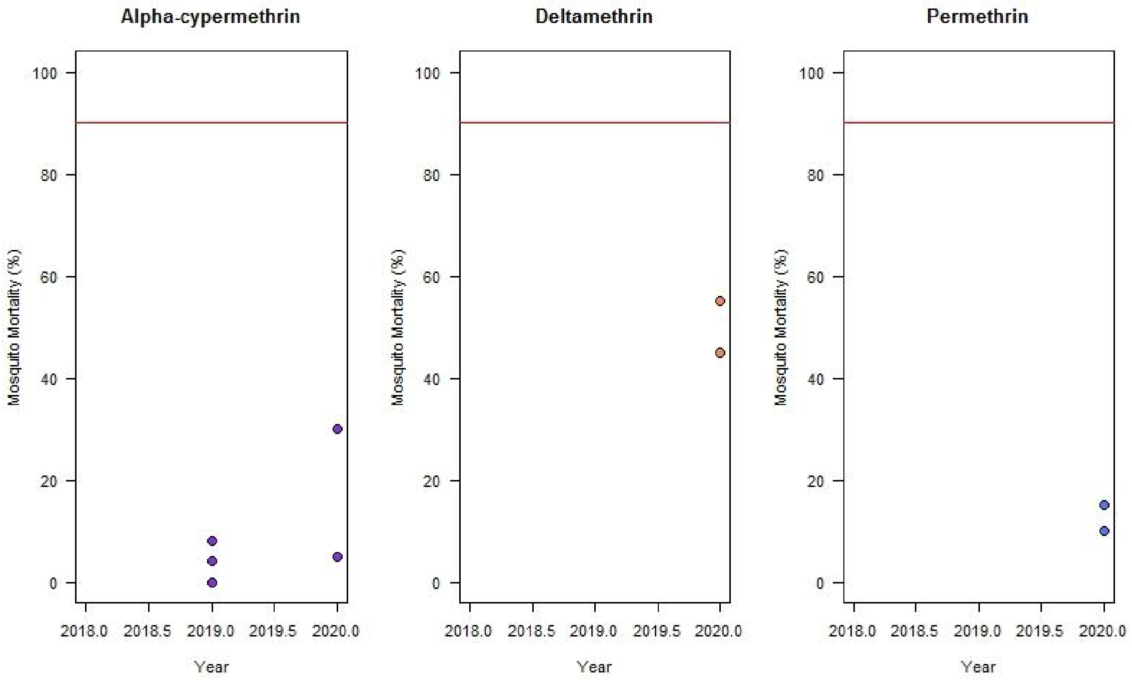

I) Tiefora (Ranson group)

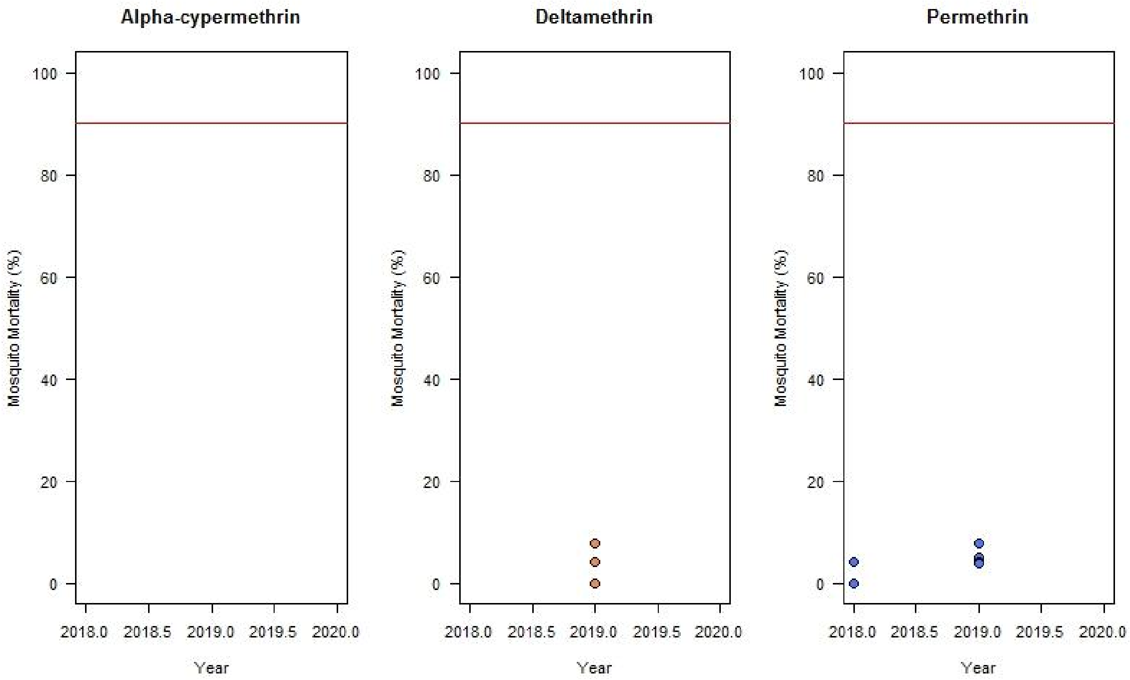

J) VK7 2014 (Ranson group)

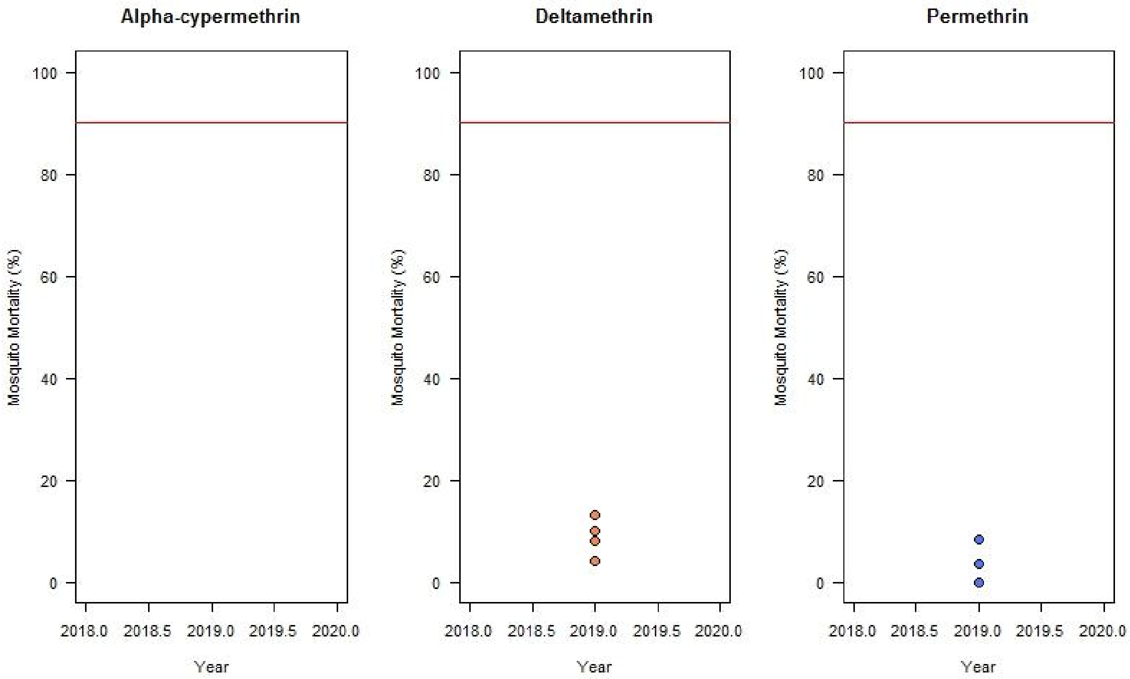

K) Kisumu (LITE)

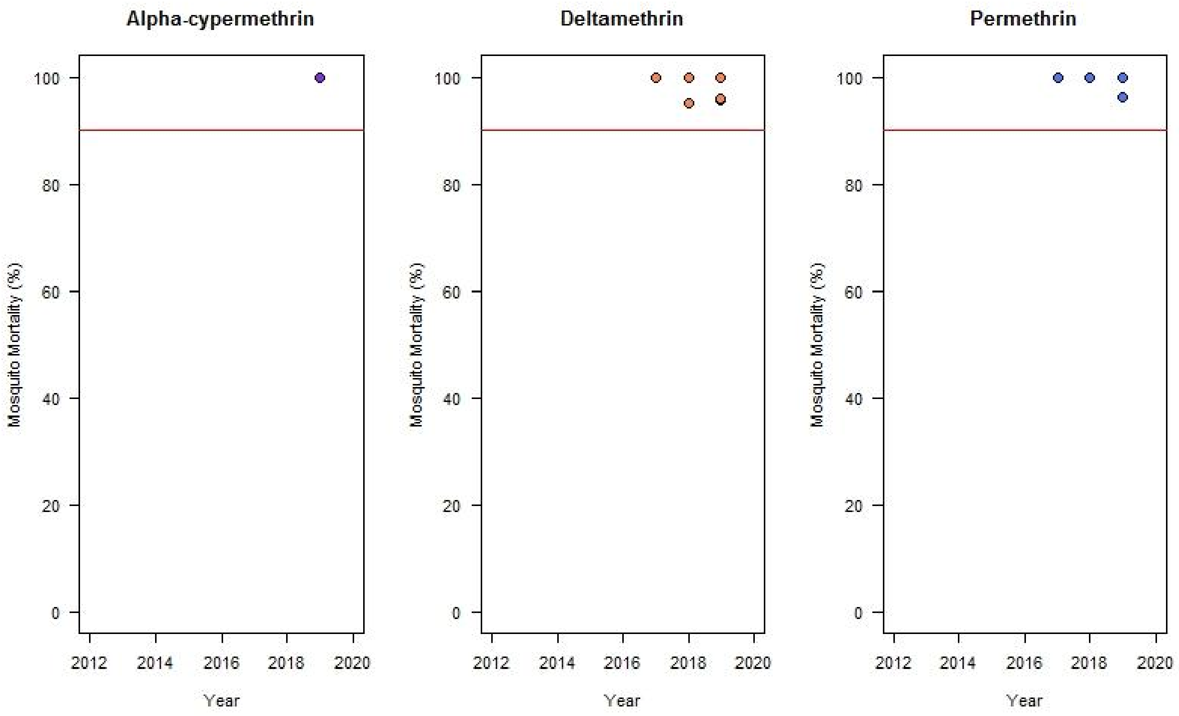

L) Moz (LITE)

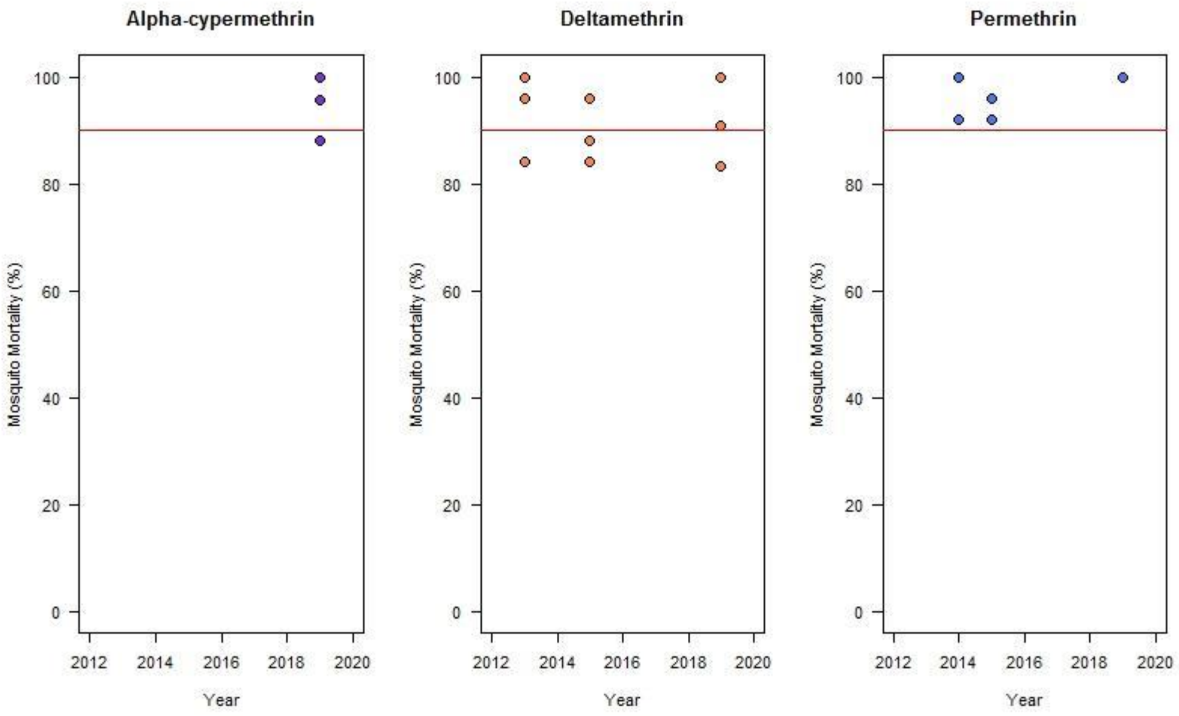

M) FUMOZ-R (LITE)

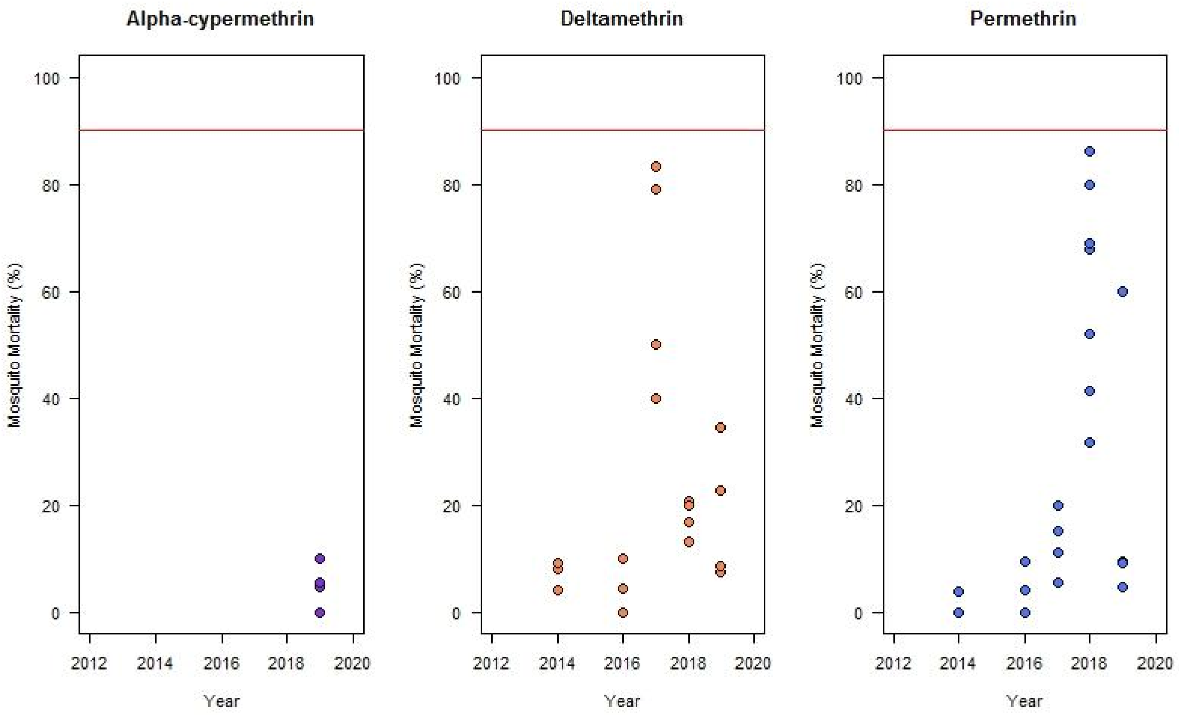

N) Tiassalé 13 (LITE)

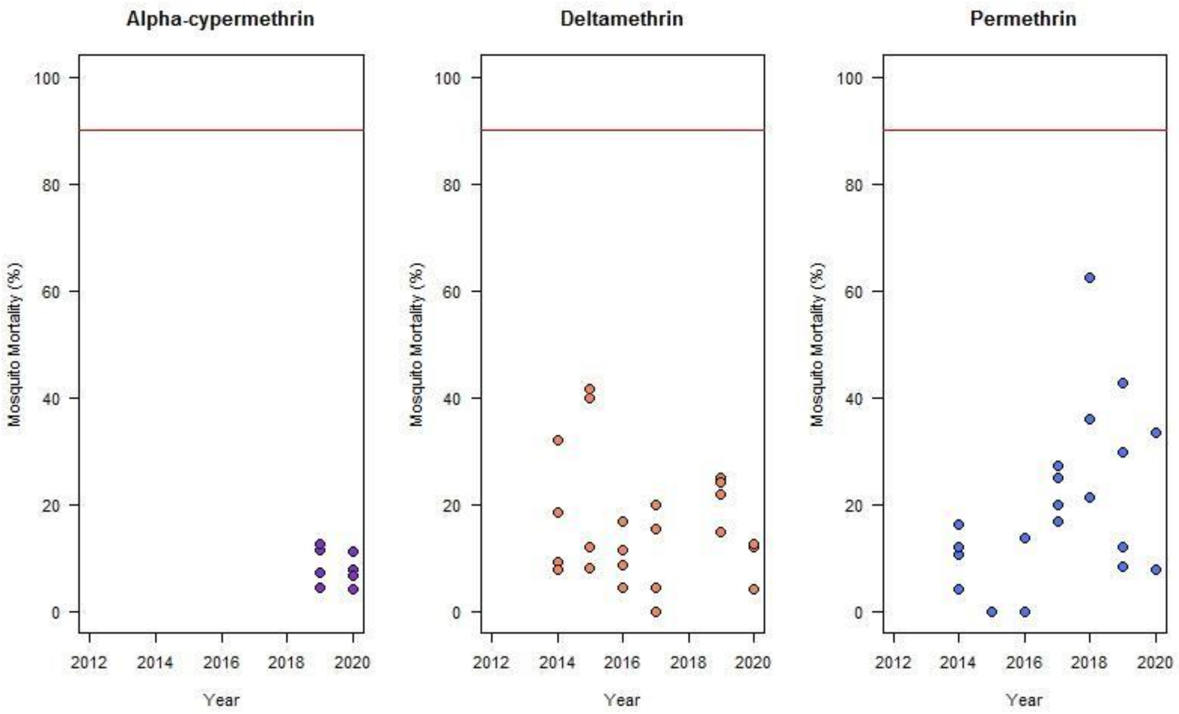

O) VK7 2014 (LITE)

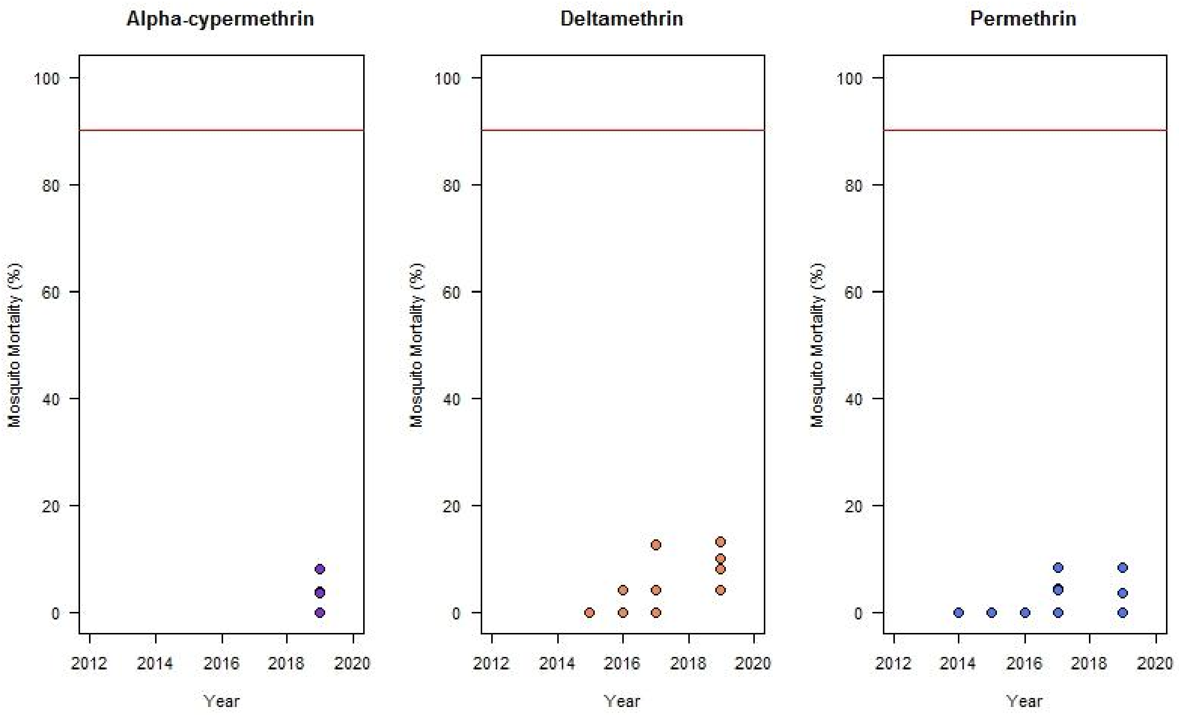

Figure S5. Mosquito mortality over time following exposure to pyrethroids in a standard WHO tube bioassay. Ranson group (**A–J**) and LITE (**K–O**) mosquito strains were exposed to deltamethrin 0.05%, permethrin 0.75% and α-cypermethrin 0.05% in a standard 1-hour WHO tube bioassay, and their 24-hour mortality was recorded. Coloured circles indicate each individual replicate tube.

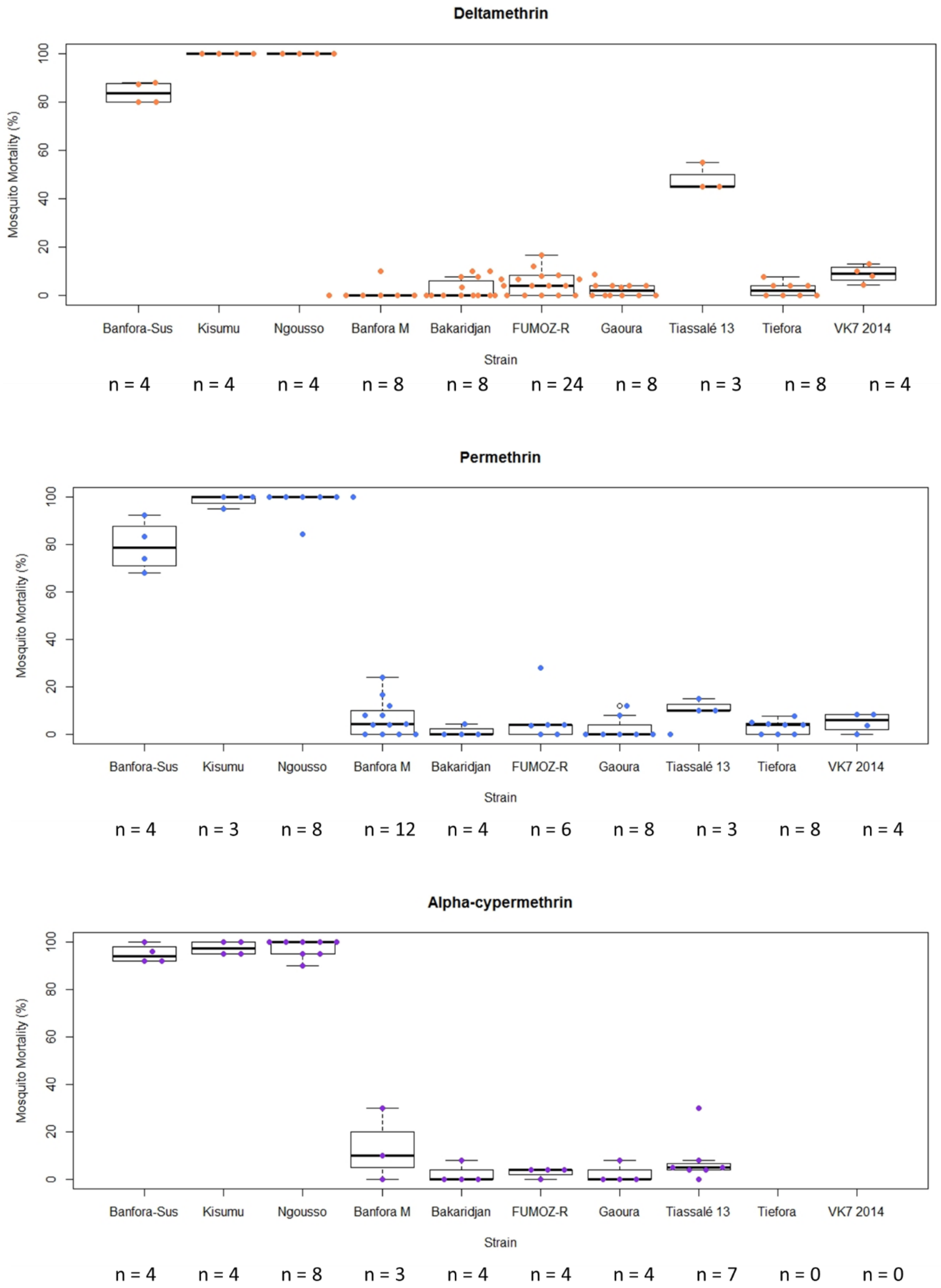

Figure S6. Box plot summarising mosquito mortality following exposure to deltamethrin 0.05% (top), permethrin 0.75% (middle), or α-cypermethrin 0.05% (bottom) in a standard WHO tube bioassay in Ranson group strains. Each box represents a different mosquito strain. Coloured circles and n-numbers indicate each individual tube replicate.

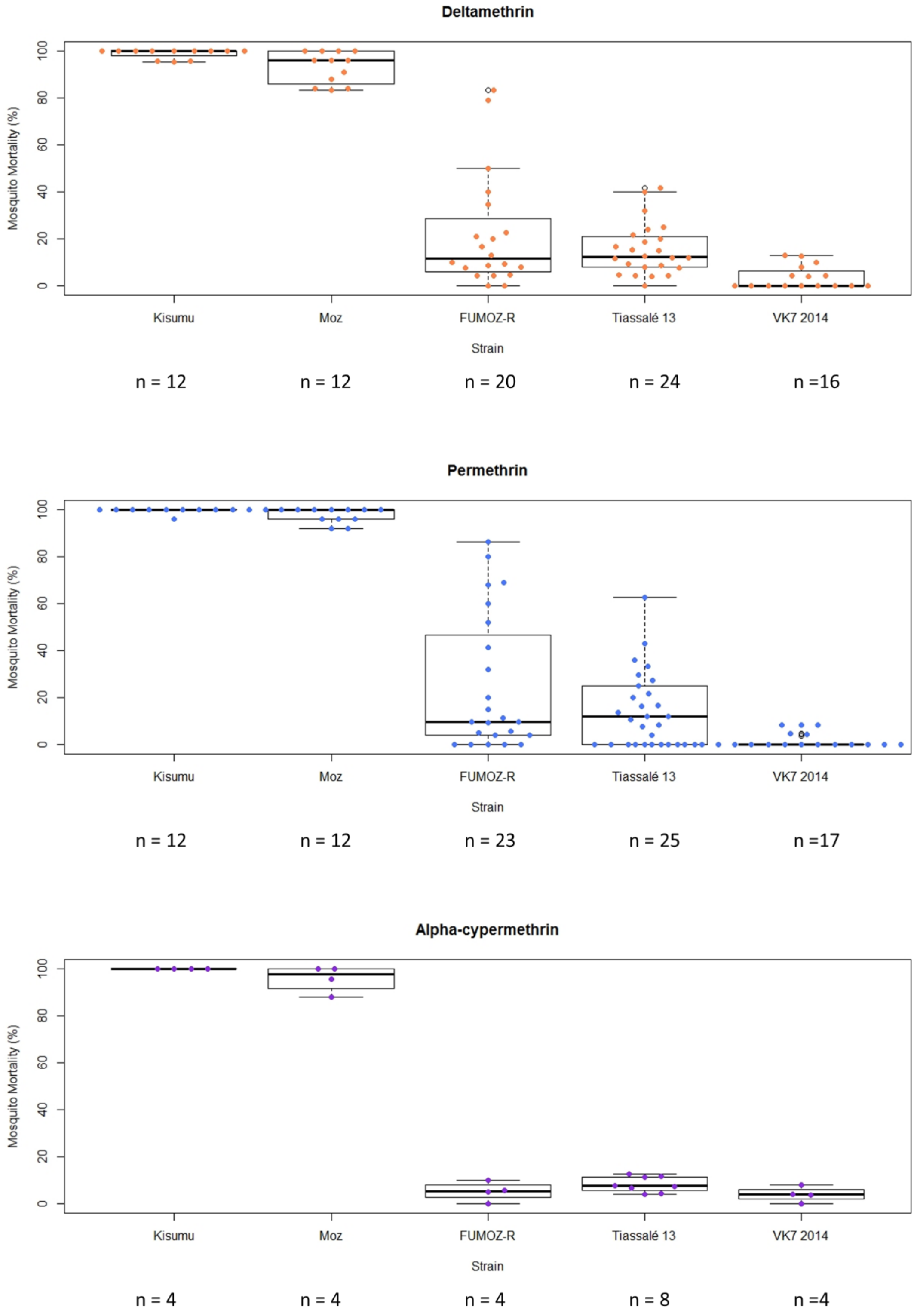

Figure S7. Box plot summarising mosquito mortality following exposure to deltamethrin 0.05% (top), permethrin 0.75% (middle), or α-cypermethrin 0.05% (bottom) in a standard WHO tube bioassay in LITE strains. Each box represents a different mosquito strain. Coloured circles and n-numbers indicate each individual tube replicate.

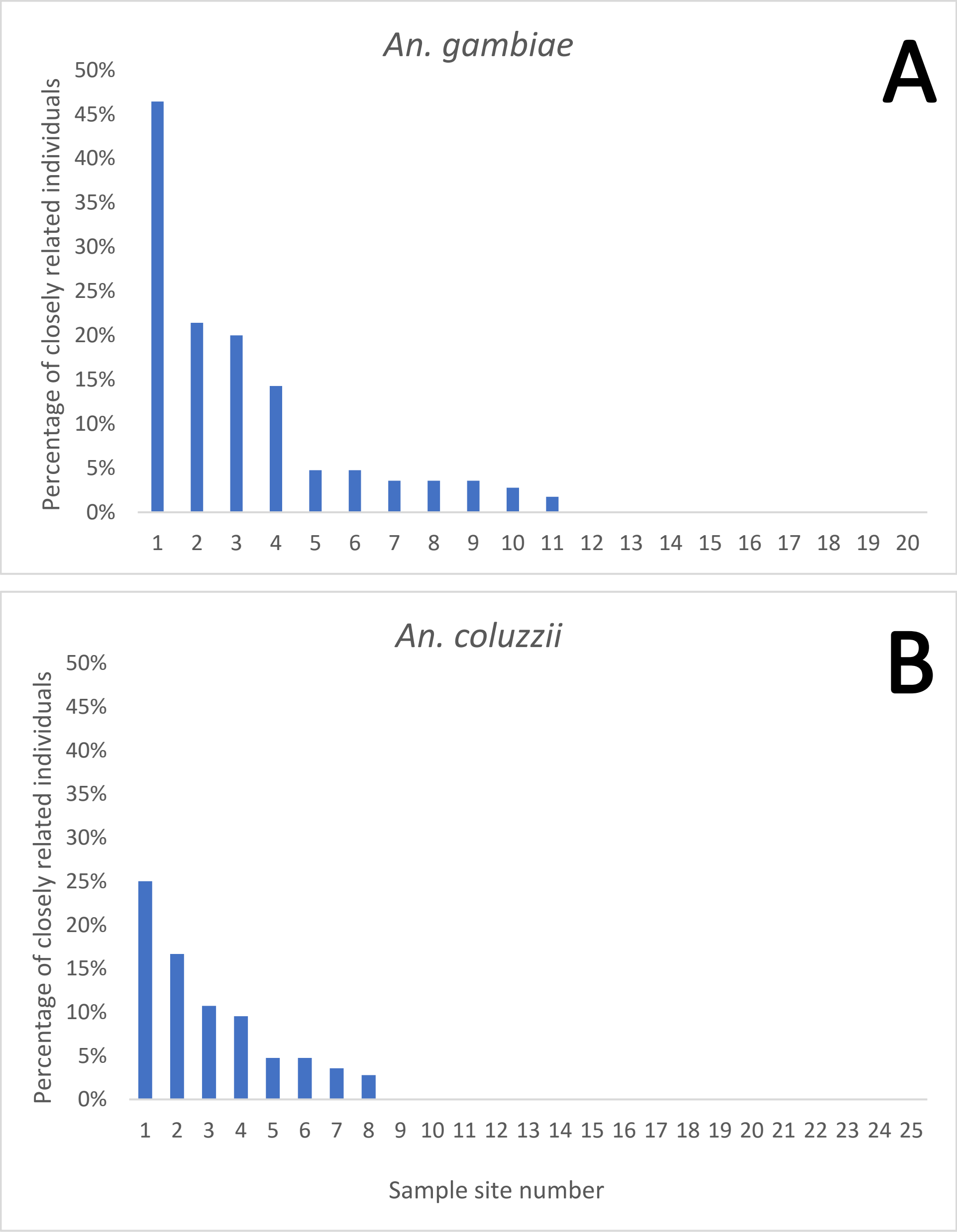

Figure S8. Distribution of larval relatedness of (**A**) *An. gambiae* and (**B**) *An. Coluzzii* within breeding sites sampled across multiple locations in 2016 from southern Ghana. Individuals (median N=7 per site) were genotyped by reduced coverage whole genome sequencing and pairwise relatedness categories estimated from chromosome 3 data (2229 SNP markers) as siblings or unrelated using ML-Relate [41]. The percentage of sibling relationships is shown. Sample site numbers are nominal and are not equivalent between plots.
